# A New Ion-Imprinted Chitosan-Based Membrane with an Azo-Derivative Ligand for the Efficient Removal of Pd(II)

**DOI:** 10.3390/ma10101133

**Published:** 2017-09-26

**Authors:** Maria Pia Di Bello, Maria Rosaria Lazzoi, Giuseppe Mele, Sonia Scorrano, Lucia Mergola, Roberta Del Sole

**Affiliations:** Department of Engineering for Innovation, University of Salento, via per Monteroni Km 1, 73100 Lecce, Italy; mariapia.dibello@unisalento.it (M.P.D.B.); mariarosaria.lazzoi@unisalento.it (M.R.L.); giuseppe.mele@unisalento.it (G.M.); sonia.scorrano@unisalento.it (S.S.); lucia.mergola@unisalento.it (L.M.)

**Keywords:** palladium, chitosan, azobenzene, ion imprinted polymer membrane, adsorption

## Abstract

Herein, we described the synthesis of a novel ion-imprinted membrane for the detection of palladium(II) prepared through the glutaraldehyde crosslinking of chitosan with a 4-[(4-Hydroxy)phenylazo]benzenesulfonic acid ligand trapped into the membrane. The imprinting technology was used to improve adsorption capacity and adsorption selectivity, and was combined with some advantages of the developed membrane, such as low cost and ease of preparation, water-friendly synthesis, and high biocompatible chitosan material. The membranes were characterized by Fourier Transform Infrared Spectroscopy (FTIR), Scanning Electron Microscopy (SEM), and Energy Dispersive X-ray Spectrometry (EDS). The results obtained showed a high swelling ratio with a maximum value of 16.4 (1640%) at pH 4 with a strong pH dependence. Batch rebinding experiments gave a maximum adsorption capacity of 101.6 mg of Pd(II) per gram of imprinted membrane. The Pd(II) adsorption behavior was well-described by a Langmuir model with a theoretical maximum adsorption capacity of 93.48 mg g^−1^, similar to the experimental one. Finally, a selectivity study versus Ag(I), Pb(II), and Fe(III) ions demonstrated a good selectivity of chitosan-imprinted membrane towards Pd(II).

## 1. Introduction

Palladium (Pd) is a precious metal that is very conductive and highly resistant to acids, heat, and corrosion. It has a great importance in industry with application in different fields, such as catalysis, jewelry, electronic components, chemical and medical devices, and automobile elements [1,2,3,4,5]. On the other hand, its little availability in nature is responsible for its high prices and for the interest of the scientific community to find novel recovery methods of palladium from waste sources.

Several traditional techniques can be used for the extraction or separation of Pd from various sources, including chemical precipitation [6], solvent extraction [7], electrochemical technologies [8], solid phase extraction or ion exchange [9], and membrane technologies [10,11]. However, all conventional methods suffer some disadvantages, especially in terms of poor selectivity and limited cost-effectiveness of the sorbents. Moreover, nowadays, a particular consideration is devoted to the design of green adsorption systems. For instance, researchers have been studying the potential of bio-derived materials to be sustainable metal recovery technologies. In this context, chitosan-modified adsorbents have gained substantial attention [12,13]. Chitosan (CS) is a cheap, nontoxic, biodegradable, and biocompatible cationic polysaccharide produced by the partial deacetylation of chitin isolated from naturally occurring crustacean shells. It mainly consists of beta (1-4)-2amino-2-deoxy-d-glucose units. CS has been widely studied for biosensors, tissue engineering, separation film, water treatment, and so on due to its good biocompatibility, biodegradability, and multiple functional groups [14]. The amine groups of the CS backbone can react with metal ions through a chelation mechanism or an ion-exchange mechanism. In some applications, such as the removal and separation of metal ions from water solutions in the environmental field, the separation materials should have insolubility in water and also good mechanical strength in acid or alkali solutions. Since chitosan has a traditionally poor mechanical stability and partial solubility in acid solution, a modification on its structure is required to enhance its mechanical stability and insolubility. This can be reached through grafting or crosslinking processes. Moreover, CS can be readily modified in various forms, such as beads, membranes, and fibers [15,16,17]. Thus, many studies have focused on the chemical modification of CS in order to successfully employ it in precious metals extraction [18,19,20].

Azo-type molecules have gained great consideration in the photoelectric field, dye printing, and detection of metal ions [21]. On the other hand, only a little attention has been dedicated to the combination of azo-derivative compounds with CS for metal detection. To the best of our knowledge, only one study has been done that reports on the preparation of an azobenzene-modified, crosslinked chitosan employed for precious metals adsorptions [20]. Wang et al. hypothesized that an azobenzene derivative may participate in the adsorption process through the coordination of an ion metal with π electrons of –N=N–. In a very recent work, a chitosan-based, molecularly imprinted membrane for the removal of 4-nitrophenol was prepared in our laboratory [22]. However, azo-based CS material has never been used in ion-imprinted polymer systems.

Starting from the experience of our research group in the field of molecularly imprinted polymers [23,24,25,26,27,28], the imprinting technology was used here to develop a new membrane for palladium uptake based on crosslinked CS containing an azo-derivative ligand: a Pd(II)-imprinted, azobenzene-modified chitosan membrane (Pd-IAzoCsM).

The general procedure of Ion-Imprinted Polymers (IIPs) preparation consists in the formation of a ligand–metal complex followed by a copolymerization in the presence of an excess of crosslinking agent. After polymerization, the template ion is removed by washing procedures, leaving within the polymer network three-dimensional recognition cavities with a predetermined orientation according to their stereochemical interaction with the template metal ion [15,29,30]. Thus, the resultant polymer recognizes and binds selectively only the template ions. The high selectivity toward the target ion is due to an ion memory imprinted in the polymer (imprinting effect) that retains selective ion information regarding both morphological and chemical properties of the template [29,31] thanks to the presence of the template during the polymerization process. The generated cavities, complementary in size and shape to the target ion, will be able to selectively bind the ion of interest even in a complex matrix-containing, structurally similar compound. Recently, a noteworthy research interest has been addressed to ion imprinting for palladium recognition [32,33]. More generally, various works in the field of metal-imprinted chitosan membranes for the purification of wastewaters have appeared in the literature very recently, proving the appeal towards these innovative and low cost systems [34,35,36,37].

Herein, the synthesis of a novel palladium-imprinted membrane based on a chitosan matrix is reported. The azo-water soluble derivative 4-[(4-Hydroxy)phenylazo]benzenesulfonic acid (PABSA) was trapped in the chitosan matrix through glutaraldehyde crosslinking with Pd(II) ions as template. The membrane was characterized by Fourier Transform Infrared Spectroscopy (FTIR), Scanning Electron Microscopy (SEM), Energy Dispersive X-ray Spectrometry (EDS), and swelling studies. The adsorption behavior of Pd (II) from water solutions with the Pd-IAzoCsM membrane was evaluated. Comparative selective studies were carried out with Ag(I), Pb(II), and Fe(III) as metals ions competitors in water systems.

## 2. Results and Discussion

### 2.1. Modified Chitosan Membranes Synthesis

PABSA was chosen as the Azo-type compound for its solubility in water, since the preparation of chitosan membranes was obtained starting from an acid water solution of CS. Glutaraldehyde (GA) is a well-known crosslinking agent, which was used in this study in order to obtain a networked chitosan membrane insoluble in water.

**Scheme 1:**
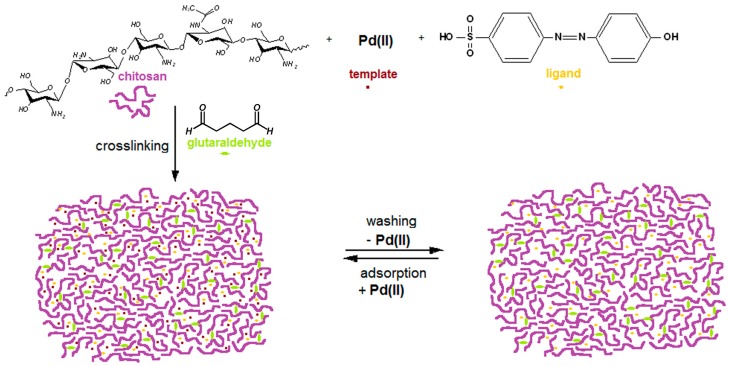
Proposed schematic process for the preparation of an imprinted Pd(II) chitosan membrane.

Various attempts were made in order to find the optimal procedure for membrane preparation, changing different experimental conditions such as concentrations, reagents ratio, temperature, and reaction time. In the adopted procedure, a concentration of chitosan of 14.2 mg mL^−1^ was used with a PABSA/chitosan molar ratio in the range of 31–120. GA was added to the reaction mixture as the last reagent, then the membrane was prepared by casting the solution onto a dish and allowing the water to evaporate at 30 °C for 5 days and the GA to crosslink the CS polymer (Scheme 1).

During the crosslinking process, PABSA ligand was trapped into the polymeric matrix along with template ions, and after the washing procedure, Pd ions were removed, as confirmed from the EDS studies described in the following paragraph. The resulting membrane, Pd-IAzoCsM, and the corresponding non-imprinted chitosan membrane (NIAzoCsM), prepared with the same method of the imprinted one but without the addition of Pd(II) ions, showed a high water insolubility typical of a crosslinked chitosan polymer and a brownish colour, which can be ascribed to the presence of the orange PABSA ligand.

**Figure 1:**
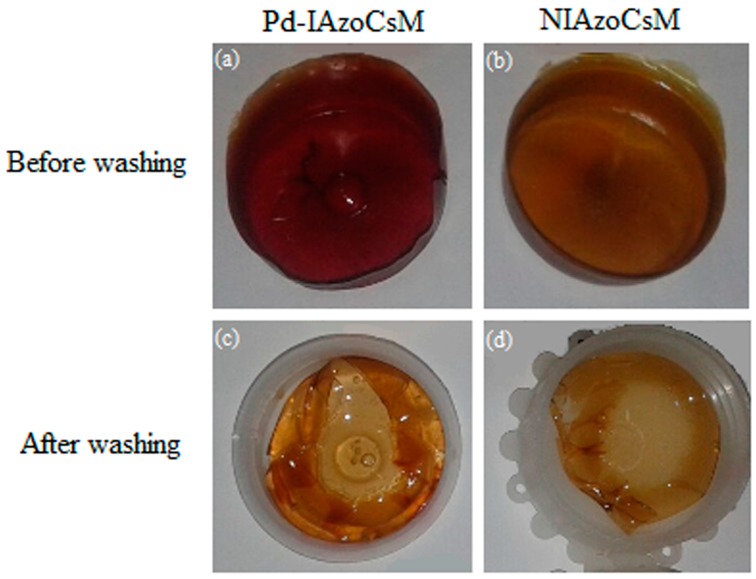
Photographs of the synthesized membranes (**a**) Pd(II)-imprinted, azobenzene-modified chitosan membrane (Pd-IAzoCsM) and (**b**) non-imprinted, azobenzene-modified chitosan membrane (NIAzoCsM) before washing in their dried state and (**c**) Pd-IAzoCsM and (**d**) NIAzoCsM after washing with HNO_3_ 0.5 M in their swollen state.

However, a clear colour difference between imprinted and non-imprinted membranes is evident before the washing step (Figure 1a,b) due to the presence of Pd(II) ions only in the imprinted membranes that disappears after washing and the removal of palladium ions (Figure 1c,d).

### 2.2. Membranes Characterization

**Figure 2:**
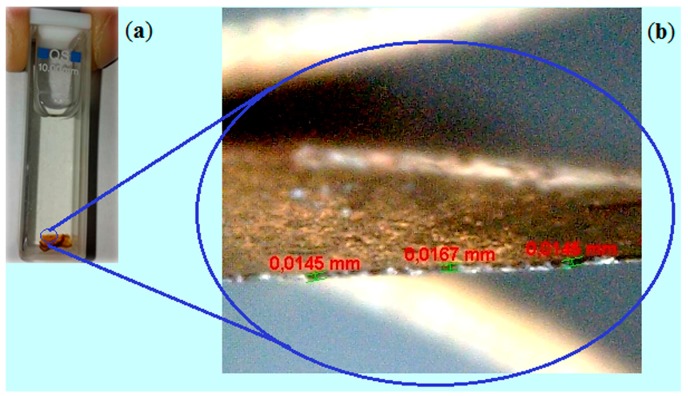
(**a**) Pd-IAzoCsM immersed in buffer solution (pH 4.1) of Pd(II); (**b**) optical photograph of Pd-IAzoCsM in a dried state.

Preliminary characterization data were thickness measurements of each membrane sample by using optical microscopy. The specimens were analysed in their dried state with a digital optical microscope obtaining with an enlargement of 230× a thickness for both membranes in the range of 13–17 μm (Figure 2b).

**Figure 3:**
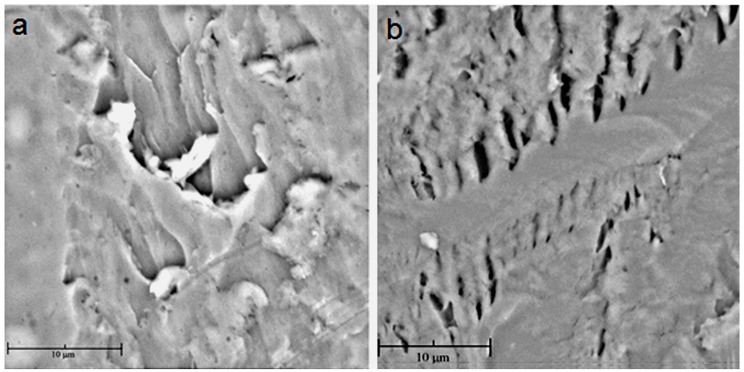
SEM images of imprinted (**a**) and non-imprinted (**b**) membranes at 8000× magnification.

**Figure 4:**
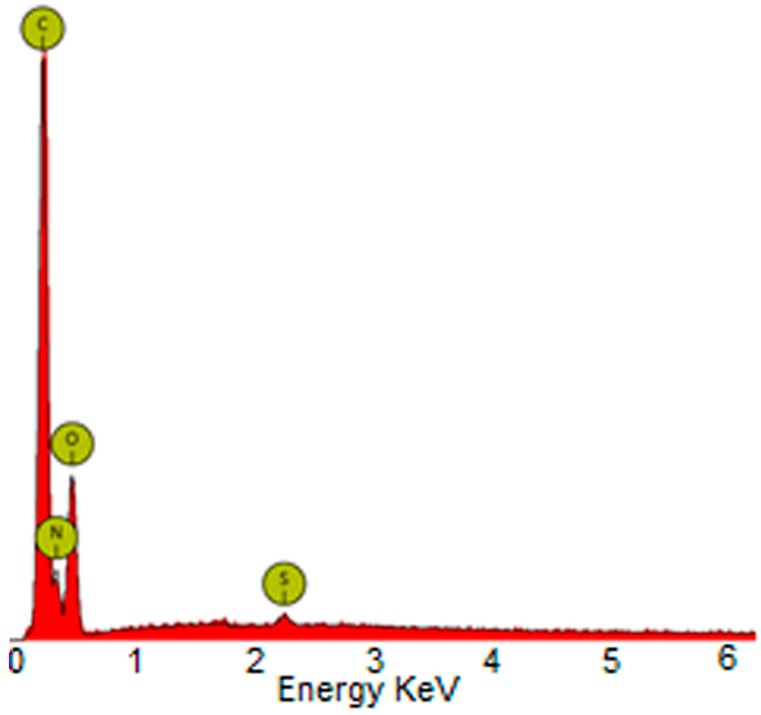
EDS of imprinted membrane Pd-IAzoCsM.

Surface Scanning Electron Microscopy (SEM) images of imprinted and non-imprinted azobenzene-modified chitosan membranes, after the washing step and having been dried in the oven, are shown in Figure 2. It can be observed that both membranes have an irregular surface structure without noticeable pores, characterized by a general roughness with some differences between them. In Figure 3a, an SEM image of Pd-IAzoCsM appears with stratified layers that are not present in the non-imprinted membrane (Figure 3b). It can be hypothesized that metal ions added during the synthesis process can affect the final membrane morphology of Pd-IAzoCsM. Moreover, to confirm azobenzene presence in the polymer membrane, an Energy Dispersive X-ray Spectrometry (EDS) analysis was performed. In Figure 4, the EDS spectrum of the imprinted membrane is reported. The atomic concentrations of carbon, oxygen, and nitrogen were 22.9%, 60.5%, and 16.2%, respectively, mostly due to the chitosan polymer matrix, while 0.5% of S confirms the inclusion of azobenzene in the polymer matrix, which is characteristic of the sulfanilic group in the azobenzene structure. Finally, palladium was not found, confirming a complete removal of Pd ions during the washing steps. As expected, similar EDS results were obtained for non-imprinted, azobenzene-modified chitosan membrane.

PdIAzoCsM was incubated with a solution of Pd(II) 100 mg L^−1^, and after drying an atomic palladium concentration of 2% was measured in the EDS of its SEM images, confirming the adsorption of the metal ions on the membrane surface.

**Figure 5:**
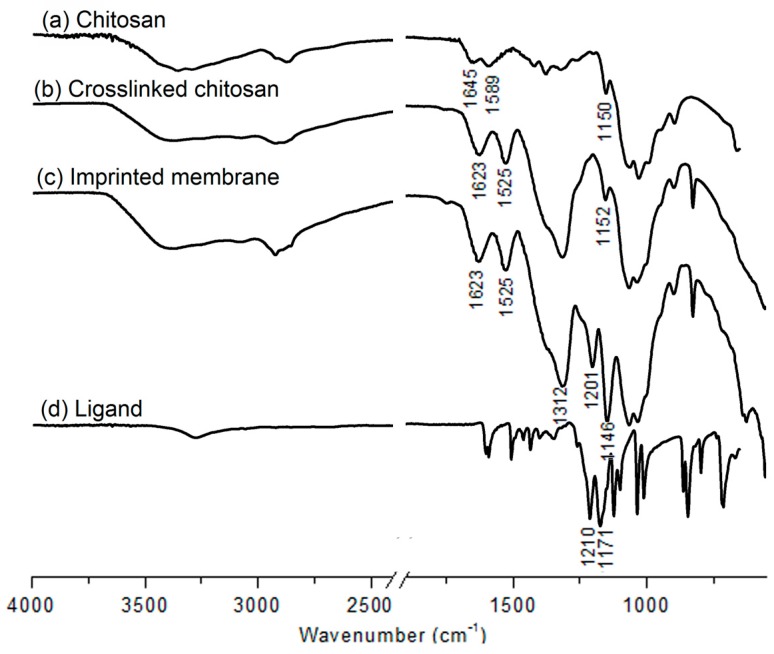
FTIR spectra of chitosan (**a**); Glutaraldehyde (GA) crosslinked chitosan (**b**); Pd-IAzoCsM (**c**); and PABSA (**d**).

For the chemical identification of the membrane, infrared spectroscopy was also used. Fourier Transform Infrared Spectroscopy (FTIR) spectra of chitosan (Figure 5a), GA crosslinked chitosan (Figure 5b), crosslinked membrane Pd-IAzoCsM (Figure 5c), and PABSA (Figure 5d) are compared in Figure 5. Figure 5a shows a typical IR spectrum of CS before crosslinking, with a peak at 3360 cm^−1^ (–OH and –NH stretching vibrations), and two bands at 2915 cm^−1^ and 2876 cm^−1^ for the C-H stretching vibration of methane in the residual-sugar group. The distinct amide I band and amide II band at 1645 cm^−1^ and 1589 cm^−1^, respectively, and the absorption bands at 1152 cm^−1^ (asymmetric stretching of C–O–C bridge), 1061 cm^−1^, and 1027 cm^−1^ (C–O stretching) are also typical of the CS saccharine structure [20,38,39]. In order to identify and discriminate from crosslinking signals and PABSA signals in the FTIR spectrum of the imprinted membrane (Figure 5c), a comparison with a glutaraldehyde crosslinked chitosan spectrum (Figure 5b) and a PABSA spectrum (Figure 5d) was made. Figure 5b shows some significant differences compared to CS (Figure 5a) at 1623 cm^−1^, 1525 cm^−1^, and 1312 cm^−1^ that can be directly ascribed to the crosslinking of the CS backbone through GA linkages, since the only difference between them was the addition of GA. The same absorbance bands were found also in the imprinted membrane (Figure 5c) that confirm GA crosslinking. Moreover, it can be noted that spectrum c appears to be quite similar to spectrum b except for the presence of two additional signals at 1210 cm^−1^ and 1171 cm^−1^. From a comparison with PABSA (Figure 5d), where it can be seen that the highest peaks are at 1210 cm^−1^ and at 1171 cm^−1^, we can confirm also the inclusion of PABSA molecules into the polymeric structure of Pd-IAzoCsM.

Finally, the non-imprinted membrane, as expected, shows a spectrum, analogous to the imprinted one, that was not shown for clarity.

### 2.3. Swelling Studies

The swelling properties of imprinted and non-imprinted membranes were investigated at various pH. Swelling ratio (SR) was measured by weighing samples before and after their immersion in water (at different pH) for about 18 h. The SR was calculated as grams of water absorbed per gram of membrane (see Equation (2) in Materials and Methods). It is well-known that the high swelling behaviour of CS in water at low pH is a consequence of the ionization of amine groups of the chitosan backbone and of the elongation of the random coil CS chains. Generally, the swelling ratio decreases after a crosslinking process, since some amine groups in chitosan are consumed due to the crosslinking reaction and are not available for ionization, and there are elastic restrictions imposed by the crosslink that prevent expansion in water [17] with values of swelling ratio lower than 2 that are almost pH invariable [40].

**Figure 6:**
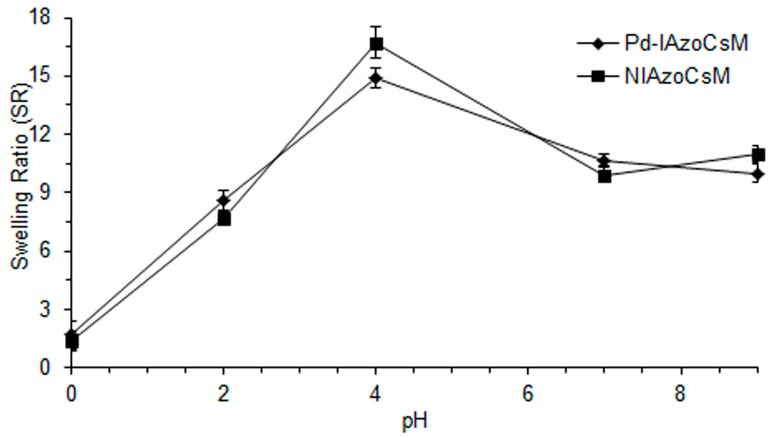
Equilibrium swelling ratio versus pH values of imprinted and non-imprinted membranes.

Figure 6 shows the swelling ratio of both imprinted (Pd-IAzoCsM) and non-imprinted (NIAzoCsM) chitosan membranes measured at different pH. Differently from the literature data, the membranes prepared in this work show a maximum Swelling Ratio (SR) of 16.4 (1640%) at pH 4 with a strong pH dependence. This confirms that the swelling behaviour is strictly related to the pH value of the solution. It is evident that the swelling ratio is much higher than the typical swelling ratio of GA crosslinked chitosan polymers (except for a pH value of 0). It can be hypothesized that the inclusion of PABSA ligand into the polymer membranes allows a significant increase of SR through its sufanilic ionizable groups, which change in turn the extent of ionic interactions with the pH value. The membrane crosslinking density could be thus changed by modifying the pH values of the environment. Another contribution to these high swelling values, despite the crosslinked nature of the membranes, could be the influence of PABSA molecules present during the crosslink process that can lead to an increased degree of swelling compared to similar GA crosslinked chitosan.

As expected, both imprinted and non-imprinted membranes share similar swelling behaviour, as they have similar chemical compositions.

To validate the PABSA influence on the swelling properties of the above-studied polymers, additional swelling experiments were conducted on a crosslinked chitosan membrane prepared similarly to the imprinted one without adding the PABSA ligand and the Pd(II) ions in the synthesis. In this case, SR values lower than 2% were measured at every pH coherently with the literature SR values of crosslinked chitosan polymers [40].

### 2.4. Adsorption Behaviour

**Figure 7:**
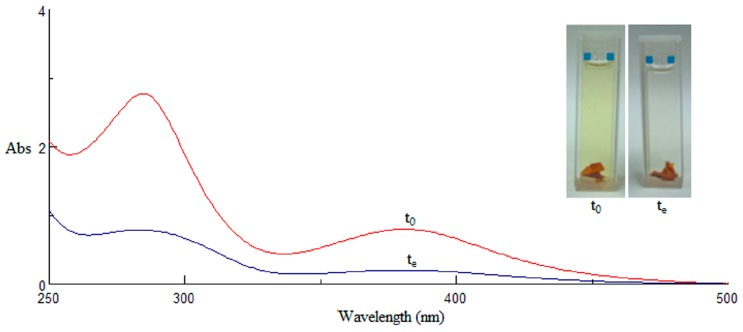
UV-visible spectra of 100 mg L^−1^ of Pd(II) solution at pH 4.1 (sodium buffer acetate 0.1 M) incubated with Pd-IAzoCsM and registered immediately after the addition of the membrane (t_0_) and at the equilibrium time, t_e_ (18 h), and their corresponding photographs.

Adsorption experiments with Pd(II) were carried out to evaluate the binding capacity of Pd-IAzoCsM and NIAzoCsM. A qualitative evaluation of palladium adsorption on the membrane was possible through the observation of a solution colour decrease when the initial pale orange Pd(II) solution was incubated with the membrane, as also confirmed from the UV visible spectra of the solution (Figure 7).

**Figure 8:**
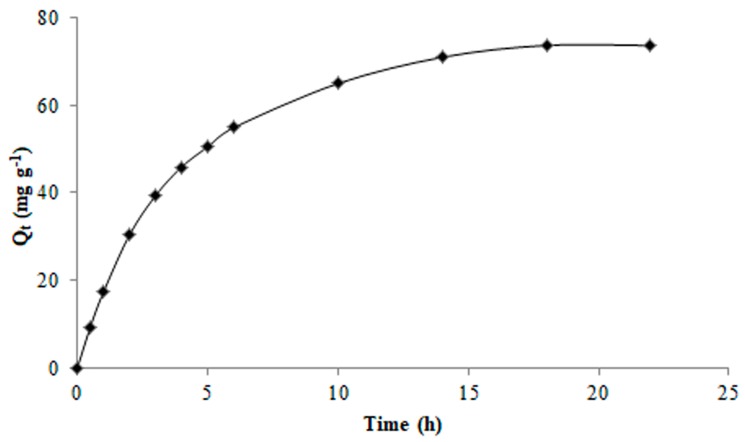
Adsorption kinetic curve of Pd-IAzoCsM.

A preliminary study was performed to find good experimental conditions for batch rebinding trials. At first, the progress of the binding process at different times was evaluated. The kinetic of adsorption that describes the Pd(II) uptake rate was performed. To do this, the imprinted CS membrane was immersed in a quartz cuvette containing a solution of 100 mg L^−1^ of Pd(II). Then, several UV-Vis measurements of the solution at different time intervals were carried out directly into the cuvette, avoiding several withdrawal and dilution steps. Figure 8 displays the kinetic curve of the Pd(II) binding capacity of chitosan membrane (*Q_t_*) as function of time. Only 17 mg g^−1^ (23%) of sorption was quantified after 1 h, this level rises up to 55 mg g^−1^ (75%) after 6 h, and reaches the plateau phase of 74 mg g^−1^ (100%) within 18 h. Thus, an equilibrium time of 18 h is considered for incubation.

Since the pH of the adsorption medium plays an important role in the uptake of the metal ions in imprinted polymer systems, different pH solutions were considered in rebinding experiments. A known concentration of 10 ppm of Pd(II) in acidic conditions was tested at pH 0.5, in buffer acetate at pH 4.1, and in buffer phosphate at pH 6.4. No alkaline solutions were used, because it is known that Pd(II) in solution at high pH can react with hydroxide ions, such as Na(OH), to precipitate as a hydrated form of PdO [32].

Considering the results achieved, we have chosen to work at pH 4.1 using sodium buffer acetate 0.1 M as binding solution. Three milligrams (3 mg) of membranes were incubated in 3 mL of buffer acetate solution of Pd(II) at different concentrations (from 5 mg L^−1^ to 330 mg L^−1^) for 18 h.

**Figure 9:**
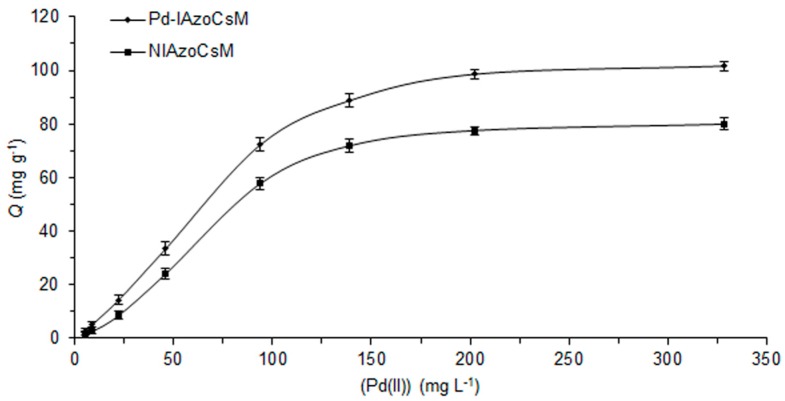
Binding isotherm of the imprinted and non-imprinted membranes.

In Figure 9, the amount of palladium adsorbed on the membranes at different concentrations is reported. The binding capacity of both membranes (*Q*) was calculated according to Equation (3) (see Materials and Methods). Pd-IAzoCsM showed a reasonably higher binding capacity than NIAzoCsM, which suggested a greater number of recognition sites and a high affinity capacity for template (Pd) on the surface of the imprinted membrane.

The graphic clearly shows that the binding capacity increases as a function of the increasing concentration of Palladium, reaching a plateau with an experimental maximum adsorption capacity of 101.6 mg g^−1^ for Pd-IAzoCsM and 80.0 mg g^−1^ for NIAzoCsM.

**Figure 10:**
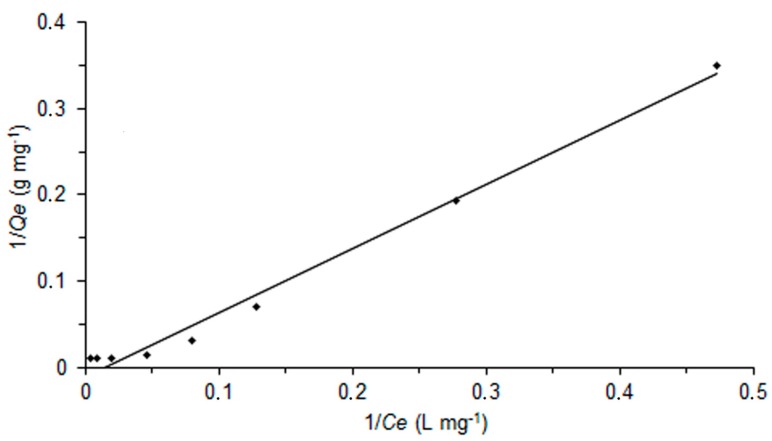
Langmuir isotherm of imprinted membrane Pd-IAzoCsM with equation *y* = 0.7431*x* − 0.0107.

The data achieved from the rebinding study were used to evaluate the binding characteristics of the imprinted polymer by using Langmuir analyses. The isotherm line, obtained from Equation (4) (see Materials and Methods), is represented in Figure 10. The Pd(II) adsorption behaviour on the membrane is well-described by a Langmuir model, as highlighted from the high R^2^ value of the Langmuir isotherm of 0.988 related to the straight line shown in Figure 10. Thus, a uniform adsorption on the membrane surface can be supposed. A Langmuir constant (KL) of 1.44 × 10^−2^ L mg^−1^ was calculated, with a maximum adsorption capacity (*Q*_max_) of 93.48 mg g^−1^, which is a theoretical value obtained from a Langmuir model similar to the experimental one.

A comparative assessment of adsorption of Pd(II) ions from various systems from the literature allows us to observe that the membrane of this work with a binding capacity of 101.6 mg g^−1^ shows higher performance than other literature systems [41,42]. Sharififard et al. reported an adsorption capacity of 62.50 mg g^−1^ with chitosan that decreased to 42.48 mg g^−1^ when activated carbon coated with chitosan was used in order to improve the mechanical and chemical properties of chitosan. Kalhor et al. prepared ion-imprinted polymer nanoparticles for selective Pd(II) recognition, and they calculated a sorption capacity of 9.4 mg g^−1^ in 30 ppm of Pd(II) solution versus a Q value of about 20 mg g^−1^ for our membrane Meanwhile, other literature systems show a moderately higher adsorption capacity [19,43]. Park et al. prepared glutaraldehyde crosslinked chitosan beads with a maximum sorption of Pd(II) in the range of 100–140 mg g^−1^. Finally, Yunus et al. designed a biomolecules–cellulose complex for palladium recovery, and found a maximum adsorption capacity of 175.44 mg g^−1^.

The membrane was also tested in a medium different from aqueous solutions in order to verify the possibility of utilizing the membrane as a sorption system when the palladium ions are dissolved in other media. Ethylene glycol was chosen as a polar organic solvent in which palladium ions have high solubility. In a quartz cuvette, 3 mg of the membrane was immersed into 3 mL of an ethylene glycol solution containing 100 mg L^−1^ of Pd(II), and the absorbance decrease of the solution at 385 nm was monitored by a UV-visible spectrometer after 18 h of incubation at room temperature. The membrane worked also in ethylene glycol with a binding capacity Q of 79 mg g^−1^, which was slightly higher of the Q value obtained from a kinetic study in aqueous solution at the equilibrium time (74 mg g^−1^).

Moreover, reusability of the adsorptive Pd(II) imprinted membrane was confirmed via three sorption–regeneration cycles using HNO_3_ 0.5 M as desorbing agent. Finally, the membrane can be stored for more than one year, at room temperature and in a dried state, without losing its adsorption properties.

### 2.5. Selectivity Studies

**Figure 11:**
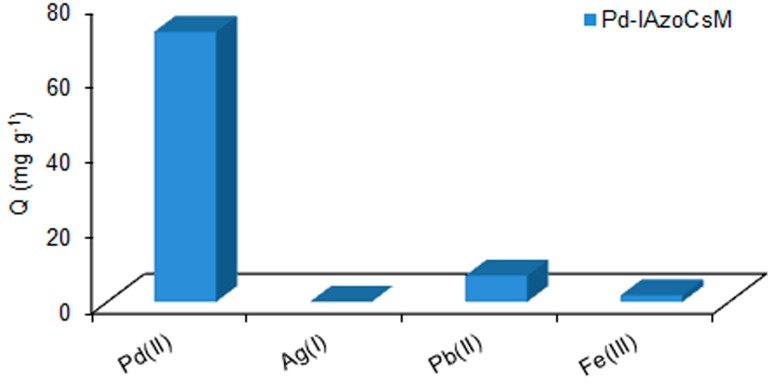
Selective adsorption behaviour of imprinted membrane Pd-IAzoCsM.

In order to evaluate the adsorption selectivity of the imprinted membrane for Pd(II) ions, a selectivity study was carried out at a concentration of 93 mg L^−1^. Common competitor ions of palladium, such as Ag(I), Pb(II), and Fe(III) ions, were chosen to evaluate selectivity performance. In Figure 11, the binding capacity (Q) of the imprinted membrane was indicated for each competitor. As it can be seen, the Pd-IAzoCSM showed significantly lower binding capacities towards Ag(I), Pb(II), and Fe(III) compared to Pd(II), thus demonstrating the good selectivity of the Pd-IAzoCSM for the template.

Nevertheless, it is known in the literature that crosslinked chitosan shows high adsorption capacities for metals such as Ag(I) [44], Pb(II) [45,46], and Fe(III) [47,48] with adsorption capacities ranging from 50 mg g^−1^ to 750 mg g^−1^; in this work, the imprinting effect conferred on Pd-IAzoCSM during the polymerization process enhances its selectivity for Pd(II), reducing considerably the adsorption of the competitor metals.

Finally, distribution coefficients (*K_d_*) and selectivity coefficients (*k*) for Pd-IAzoCsM and NIAzoCsM were also calculated along with relative selectivity coefficients (*k*′) of Pd template ions versus their competitors. It is worth noting that the distribution coefficient of palladium ions for the imprinted membrane is 2.17 times higher than that of the non-imprinted membrane. This result suggests that Pd-IAzoCsM shows more specific binding sites for Pd(II) than NIAzoCsM. The selectivity coefficients (*k*) of the imprinted membrane for Pd(II) with respect to the competitor species Ag(I), Pb(II), and Fe(III) were 2780.0, 42.3, and 182.7, respectively. These high values confirm a good selectivity of the Pd-IAzoCsM for Pd(II).

## 3. Materials and Methods

### 3.1. Materials and Apparatus

CS (degree of deacetylation 75–85%, low molecular weight, 50,000–190,000 Da), acetic acid (99.8%), and Palladium(II) acetate were purchased from Sigma-Aldrich (Steinheim, Germany). GA (50 wt % content in distilled water) and ethylene glycole were supplied by Fluka (Steinheim, Germany). Analytical grade acetonitrile (MeCN) was obtained from J.T. Baker (Deventer, The Netherlands). Sodium acetate was supplied from Carlo Erba (Milano, Italy). All chemicals were used without further purification. PABSA was prepared according to our previously reported procedure [22].

Elemental standard solutions of Pd(II), Pb(II), Fe(III), and Ag(I) were prepared by an appropriate dilution of 1000 mg·L^−1^ stocks purchased from Fluka (Steinheim, Germany). Nitric acid (>65%) and chloridric acid (>37%) for trace metal analysis were purchased from Sigma Aldrich (Steinheim, Germany) Deionized water was provided by a water purification system (Human Corporation, Seul, Korea). Batch rebinding experiments were carried out using a Jasco V-660 UV-visible spectrophotometer (Jasco, Palo Alto, CA, USA).

A rocking table type Rotamax 120 from Heidolph Instruments (Schwabach, Germany) was used for shaking incubated membranes. Sonication was carried out using a Sonorex RK 102H ultrasonic water bath from Bandelin Electronic (Berlin, Germany). Centrifugation was achieved with a PK121 multispeed centrifuge from Thermo Electron Corporation (Château Gontier, France).

SEM and EDS analyses were recorded with a Phenom ProX microscope (Phenom-World B.V., Eindhoven, Germany), at an accelerating voltage of 10 kV. Membranes were analysed (at magnification 8000) to record the morphology of membranes’ surface. For each membrane, SEM images of three samples were carried out recording three SEM images for each sample. An EDS spectrum of each SEM image was acquired with 56,000 counts in 30 s. EDS atomic concentrations were calculated as the average of all the analyses on each membrane. The thickness of the membranes was measured using a Dino-Lite digital microscope (AnMo Electronics Corporation, Taipei, Taiwan). FTIR analyses were recorded on a Perkin Elmer Spectrum (PerkinElmer, Inc., Waltham, MA, USA) One FTIR spectrometer equipped with an MCT-A detector. The internal reflection element (IRE) was a three-bounce 4 mm diameter diamond microprism mounted into a stainless steel plate. The absorption spectra of membranes were recorded in the wavenumber range of 4000–650 cm^−1^ by cumulating 32 scans at a resolution of 4 cm^−1^. For pH measurements, pH meter Basic 20, (Crison, Alella, Barcelona, Spain, Europe,) was used. Ions quantification in the batch rebinding experiments and selectivity experiments was achieved by Thermo Scientific Inductively Coupled Plasma Mass spectrometry (ICP-MS) iCAP Q ICP-MS (Thermo Fisher, Scientific, Waltham, MA, USA).

### 3.2. Preparation of Pd(II)-Imprinted, Azobenzene-Modified Chitosan Membrane (Pd-IAzoCsM)

PABSA was prepared according to our previously reported procedure [22]. CS solution was prepared by dissolving 85.2 mg of CS into 3 mL of aqueous acetic acid solution (1% *v*/*v*) and stirred until complete dissolution. PABSA solution was prepared by dissolving 15 mg of PABSA in 3 mL of aqueous acetic acid solution (1% *v*/*v*) and stirred at 40 °C till dissolution, then 2.4 mg of Pd(OAc)_2_ were added and kept at 40 °C for 1 h. PABSA solution was finally added to the CS solution and stirred at 40 °C for 1/2 h. GA solution (25%) in the amount of 17.1 μL was added to the reaction mixture under stirring, and just before gel formation a membrane was prepared by casting 2 mL of the solution onto a polypropilene dish and allowing the water in the casting membrane to evaporate at 30 °C for 5 days. The membrane was immersed in water three times to eliminate any unreacted GA, and several washings with water solution of HNO_3_ 0.5 M were made to remove Pd(II) from the membrane until the Pd(II) UV signal at 283 nm disappeared. Final washings of the membrane in water allowed us to remove acid traces. The membrane was dried at 60 °C overnight, giving a brownish membrane with a yield of about 75%.

NIAzoCsM as control membrane was also prepared with the same above-mentioned method, but without the addition of Pd(II) ions.

### 3.3. Swelling Studies

Equilibrium swelling measurements for imprinted, non-imprinted, and crosslinked chitosan membranes (the last membrane was prepared with the same procedure of the imprinted one without adding PABSA ligand and Pd(II) ions) were carried out immersing 1 mg mL^−1^ of membrane in water at different pH (0, 2, 4.1, 7, and 9) adjusted with acid or base. The systems were kept at room temperature for 18 h to achieve the swelling equilibrium. After being fully hydrated, the membranes were taken out and the excess water on their surface was gently removed by filter paper. The weight of the swollen membrane was measured using a Sartorius microbalance (sensitivity of 10–4 g). The swelling ratio (SR) was measured by weighing samples before and after their immersion in water for about 18 h. The SR was calculated by the following equation:

(1) $$\mathrm{SR}=\frac{W_{S}-W_{d}}{W_{d}}$$

where *W_s_* is the weight of the swollen membrane, and *W_d_* is the weight of the dried sample [49]. Thus, SR was calculated as grams of water absorbed per gram of membrane.

### 3.4. Adsorption Kinetic

The kinetic adsorption experiment was conducted as follows: 3 mg of the imprinted membrane was immersed into a quartz cuvette with 3 mL of a sodium acetate buffer solution 0.1 M (pH 4.1) raketscontaining 100 mg L^−1^ of Pd(II), and kept under constant stirring at room temperature. The concentration of Pd(II) in solution was monitored using a UV-visible spectrophotometer at different times (*t*) from *t* 0 until 22 h, following the absorbance at 381 nm (taking care to not let the beam interface with the membrane deposited on the bottom of the cuvette). The amount of Pd(II) adsorbed at time *t*, *Q_t_* (mg g^−1^), was determined from Equation (2) by the difference between the initial concentration of Pd(II) in the solution (*C_i_*) at *t* = 0 and the residual concentration at t adsorption time (*C_t_*), as:

(2) $$Q_{t}=\left(C_{i}-C_{t}\right)\frac{V}{m}$$

where *V* is the volume of incubation solution (L), and *m* is the weight of the membrane (g).

### 3.5. Batch Adsorption Experiments

The binding capacity of the imprinted membrane Pd-IAzoCsM and of its control NIAzoCsM was evaluated through batch adsorption experiments. The membrane was immersed in a glass tube to a known concentration of Pd(II) in sodium acetate buffer solution 0.1 M (pH 4.1). In all of the experiments, the membrane concentration was kept constant at 1 mg mL^−1^ with an initial Pd(II) concentration in the range of 5–330 mg L^−1^. The mixture was shaken on a rocking table for 18 h at room temperature, then the solution was filtered through a 0.20 μm porosity filter and analyzed by ICP-MS. Binding capacity, mg of Pd(II) per gram of membrane, was calculated according to the following equation:

(3) $$Q=\left(C_{i}-C_{e}\right)\frac{V}{m}$$

where *C_i_* and *C_e_* are the initial and the equilibrium concentrations of the ions, respectively (mg L^−1^), *V* is the volume of the buffer solution (L), and *m* is the weight of the membrane (g).

The adsorption isotherm is fitted with a Langmuir model. The Langmuir isotherm equation is represented in the linear form according to the following equation:

(4) $$\frac{1}{Q_{e}}=\frac{1}{\left(Q_{\max}-C_{e}K_{L}\right)}+\frac{1}{Q_{\max}}$$

where *Q_e_* is the amount of palladium ions adsorbed at equilibrium (mg g^−1^). *Q*_max_ (mg g^−1^) and *K_L_* are the maximum adsorption capacity and the Langmuir constant (L mg^−1^), respectively, that will be determined from the linear plot of 1/*Q_e_* against 1/*C_e_*.

The binding processes and measurements were performed in triplicate.

### 3.6. Selectivity Studies

A selective adsorption study of Pd-IAzoCsM versus other competitive ions was performed. In particular, solutions (93 mg L^−1^) of Pd(II), Ag(I), Pb(II), and Fe(III) were used for batch experiments, and 1 mg of membrane was incubated in 1 mL of each solution. After 18 h of incubation time, when the binding equilibrium was reached, the concentrations of Pd(II), Ag(I), Pb(II), and Fe(III) in the solutions were determined by means of ICP-MS after filtration through 0.20 μm.

The distribution coefficients of Ag(I), Pb(II), and Fe(III) were calculated by Equation (5):

(5) $$K_{d}=\frac{Q_{e}}{C_{e}}$$

where *K_d_* (L g^−1^) indicates the distribution coefficient; *Q_e_* (mg g^−1^) is the equilibrium binding amount, and *C_e_* (mg L^−1^) is the equilibrium concentration.

The selectivity coefficient of a membrane for Pd(II) with respect to the competitor species (B) can be obtained from the equilibrium binding data according to Equation (6):

(6) $$k=\frac{K_{d}\left(\mathrm{Pd}\left(\mathrm{II}\right)\right)}{K_{d}\left(B\right)}$$

where *k* represents the selectivity coefficient, and B is an Ag(I), a Pb(II), or an Fe(III) ion. The value of *k* gives an assessment of selectivity of the Pd-IAzoCsM for Pd(II). A relative selectivity coefficient *k*′ is defined in Equation (7), and the value of *k*′ can indicate the enhanced extent of binding affinity and selectivity of an imprinted membrane for the template versus a non-imprinted membrane.

(7) $$k^{\prime }=\frac{k_{\mathrm{Pd}-\mathrm{IAzoCsM}}}{k_{\mathrm{NIAzoCsM}}}$$

where *k*_Pd-IAzoCsM_ and *k*_NIAzoCsM_ are the selectivity coefficients of the imprinted membrane and the non-imprinted membrane for Pd ions, respectively.

## 4. Conclusions

For the first time, we described the synthesis of an ion-imprinted membrane for the uptake of palladium prepared through the glutaraldehyde crosslinking of chitosan with a 4-[(4-Hydroxy)phenylazo]benzenesulfonic acid ligand trapped into the membrane. SEM images of the imprinted and non-imprinted azobenzene-modified chitosan membranes showed irregular surface structures for both polymers. EDS results and FTIR spectra confirmed azobenzene ligand inclusion in the polymer network.

The membranes prepared in this work showed a maximum swelling ratio of 16.4 (1640%) at pH 4 with a strong pH dependence. It can be hypothesized that the inclusion of PABSA ligand into the polymer membranes allows a significant increase of SR through its suphanilic ionizable groups, which change in turn the extent of ionic interactions with the pH value.

Batch rebinding experiments of palladium(II) gave a maximum adsorption capacity of 101.6 mg g^−1^ for Pd-IAzoCsM and 80.0 mg g^−1^ for NIAzoCsM. The Pd(II) adsorption behaviour on the membrane was well-described by a Langmuir model assuming a uniform adsorption on the membrane surface. Finally, a selectivity study versus Ag(I), Pb(II), and Fe(III) competitor ions demonstrated a good selectivity of Pd-IAzoCSM towards Pd(II).

Some advantages of the synthesized membrane are a low cost and ease of preparation and a high biodegradability of chitosan, which make it convenient to apply in separation processes with good feasibility for future progress in the membrane field. In this context, to evaluate the polymer membrane as a filtration device, more trials need to be realized. For instance, permeation experiments could be carried out in which the ion-imprinted membrane is placed between two rooms filled with two different solutions, a feed solution and a stripping phase.
